# Antimicrobial Activity of the Manganese Photoactivated Carbon Monoxide-Releasing Molecule [Mn(CO)_3_(tpa-κ^3^*N*)]^+^ Against a Pathogenic *Escherichia coli* that Causes Urinary Infections

**DOI:** 10.1089/ars.2015.6484

**Published:** 2016-05-10

**Authors:** Mariana Tinajero-Trejo, Namrata Rana, Christoph Nagel, Helen E. Jesse, Thomas W. Smith, Lauren K. Wareham, Michael Hippler, Ulrich Schatzschneider, Robert K. Poole

**Affiliations:** ^1^ Department of Molecular Biology and Biotechnology, The University of Sheffield, Sheffield, United Kingdom.; ^2^ Institut für Anorganische Chemie, Julius-Maximilians-Universität Würzburg, Würzburg, Germany.; ^3^ Department of Chemistry, The University of Sheffield, Sheffield, United Kingdom.

## Abstract

***Aims:*** We set out to investigate the antibacterial activity of a new Mn-based photoactivated carbon monoxide-releasing molecule (PhotoCORM, [Mn(CO)_3_(tpa-κ^3^*N*)]^+^) against an antibiotic-resistant uropathogenic strain (EC958) of *Escherichia coli.* ***Results:*** Activated PhotoCORM inhibits growth and decreases viability of *E. coli* EC958, but non-illuminated carbon monoxide-releasing molecule (CORM) is without effect. NADH-supported respiration rates are significantly decreased by activated PhotoCORM, mimicking the effect of dissolved CO gas. CO from the PhotoCORM binds to intracellular targets, namely respiratory oxidases in strain EC958 and a bacterial globin heterologously expressed in strain K-12. However, unlike previously characterized CORMs, the PhotoCORM is not significantly accumulated in cells, as deduced from the cellular manganese content. Activated PhotoCORM reacts avidly with hydrogen peroxide producing hydroxyl radicals; the observed peroxide-enhanced toxicity of the PhotoCORM is ameliorated by thiourea. The PhotoCORM also potentiates the effect of the antibiotic, doxycycline. ***Innovation:*** The present work investigates for the first time the antimicrobial activity of a light-activated PhotoCORM against an antibiotic-resistant pathogen. A comprehensive study of the effects of the PhotoCORM and its derivative molecules upon illumination is performed and mechanisms of toxicity of the activated PhotoCORM are investigated. ***Conclusion:*** The PhotoCORM allows a site-specific and time-controlled release of CO in bacterial cultures and has the potential to provide much needed information on the generality of CORM activities in biology. Understanding the mechanism(s) of activated PhotoCORM toxicity will be key in exploring the potential of this and similar compounds as antimicrobial agents, perhaps in combinatorial therapies with other agents. *Antioxid. Redox Signal.* 24, 765–780.

## Introduction

Carbon monoxide has a concentration-dependent biological activity and can act as a toxic gas and biological signaling molecule (35, 43). CO, whether endogenously applied or generated by heme oxygenases (HO) in animals, plants, and pathogenic microorganisms (5, 62), exerts potent beneficial effects on vasodilation and inflammation (35, 37, 43) and promotes phagocytosis and bacterial clearance in sepsis (8, 47). Carbon monoxide-releasing molecules (CORMs) largely circumvent the problems of delivering CO gas in the laboratory and clinic (42). For example, CORMs have potential in the treatment of infectious diseases, ischemia-reperfusion injury or multiple sclerosis (3, 14). Although many CORMs are available for biological use, most studies have used Ru-based CORMs that exhibit multispecies antibacterial activity (11, 73); however, our limited understanding of the modes of CORM action and the role of the metal and released CO hampers progress.

Innovation It is essential to define carbon monoxide-releasing molecule (CORM) toxicity if site-specific and time-controlled release of CO is to be exploited. We report a detailed characterization of the toxicity of a photoactivable carbon monoxide-releasing molecule ([Mn(CO)_3_(tpa-κ^3^*N*)]^+^) to a uropathogenic *E. coli*. Although extracellular light-driven CO release results in bacterial toxicity and respiratory inhibition, we here identify Mn-dependent hydroxyl formation in the presence of hydrogen peroxide as a critical factor. Models of CORM toxicity that invoke generation of reactive oxygen species, membrane damage, or accumulation of the metal center are not supported. Such insights open the way for new compound design and novel, clinical combinatorial therapies.

**FIG. 1.**
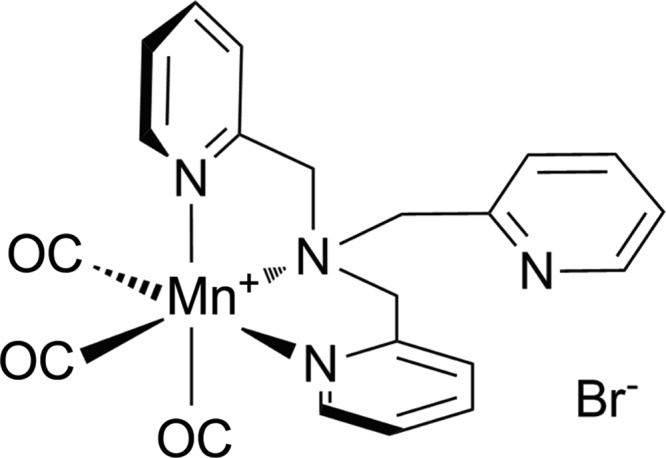
**Structure of PhotoCORM [Mn(CO)_3_(tpa-κ^3^*N*)]^+^Br^−^.** PhotoCORM, photoactivable carbon monoxide-releasing molecule.

[Mn(CO)_3_(tpa-κ^3^*N*)]Br is a novel water-soluble photoactivatable carbon monoxide-releasing molecule (PhotoCORM) stable in solution in the dark that releases CO on illumination at 365 nm (44) (Fig. 1). It is toxic to *Escherichia coli* K-12 on photoactivation, but not in dark cultures. Growth inhibition on a non-fermentable carbon source after activating the PhotoCORM *in situ*, together with the observation of CO binding to terminal oxidases, suggested that the mechanism of action of this PhotoCORM is attributable, at least in part, to the inhibition of respiration by CO (44).

Urinary tract infections (UTIs), caused predominantly by uropathogenic *E. coli* (UPEC), are the most common bacterial infections acquired outside the clinic. High prevalence, recurrence, and related morbidities are aggravated by the emergence of antibiotic resistance (13). *E. coli* 131 (ST131) is a multidrug-resistant UPEC associated with the increasing prevalence worldwide of UTIs and blood stream infections, linked with the spread of extended-spectrum β-lactamase (6) and resistance to fluoroquinolones, aminoglycosides, trimethoprim–sulfamethoxazole, and carbapenems (48). The genome of the clinically isolated uropathogen, *E. coli* EC958, a multidrug-resistant O25b:H4 strain (66), contains a number of putative virulence factors, including siderophore receptors, autotransporters, and genes conferring resistance to ciprofloxacin and other antibiotics (66).

We hypothesized that in contrast to Ru-based CO-releasing molecules such as widely used CORM-2 and −3 (71), Mn carbonyl complexes might avoid toxicity issues unrelated to the released CO alone. Furthermore, the ability to activate the compound on demand could allow controlled CO release in clinical settings using photoactivated chemotherapy (PACT) *via* catheter light guides (15, 41).

We therefore report the first study of the action of a PhotoCORM against a bacterial pathogen. We investigate the effects of CO released from the PhotoCORM on respiration, assess the fate of CO and the Mn ion when the PhotoCORM is activated in the presence of cells, and demonstrate synergy with antibiotic activity. We also report associated transcriptional changes of genes implicated in membrane integrity and metal transport, respiration, and oxidative stress. Finally, we show that PhotoCORM reacts with hydrogen peroxide (H_2_O_2_) to give hydroxyl radicals, enhancing toxicity.

## Results

### Activation of PhotoCORM in cultures of pathogenic *E. coli* EC958

The antimicrobial effect of [Mn(CO)_3_(tpa-κ^3^*N*)]^+^ is illumination dependent (44). However, since UV itself is antimicrobial (20), we first determined the optimum activation time for PhotoCORM toxicity, without damage caused by UV (Supplementary Fig. S1A, B; Supplementary Data are available online at www.liebertpub.com/ars). We found photoactivation for 6 min to be appropriate and this was used for all further experiments unless stated otherwise.

### Detection and quantification of CO release from the PhotoCORM and preparation of CO-depleted PhotoCORM control molecules

**FIG. 2.**
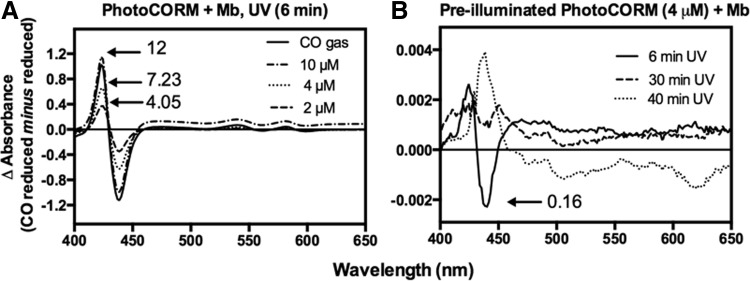
**Detection and quantification of CO release from PhotoCORM using ferrous Mb.** A stock solution of Mb (12 μ*M*) was reduced with sodium dithionite. In **(A)**, increasing concentrations of PhotoCORMs were added to myoglobin, followed by exposure to UV light (365 nm) for 6 min, and the difference in absorbance was plotted (globin plus PhotoCORM *minus* globin, all reduced). Reduced Mb was bubbled with CO gas and plotted as a control. In **(B)**, PhotoCORM (1 m*M*) was exposed to UV for increasing periods of time while stirring and then added to reduced Mb. Difference spectra were obtained as in **(A)**. Numbers with *arrows* in the graphs correspond to the concentrations of CO-Mb (μ*M*) formed by the addition of PhotoCORM or CO gas. Plots are representative of three independent repetitions. Mb, myoglobin.

To measure CO release from activated PhotoCORM in the presence of biological targets, dithionite-reduced myoglobin (Mb, 12 μ*M*) was illuminated in the presence of PhotoCORM. CO difference spectra were plotted by subtracting the spectrum of the reduced Mb from the CORM- or CO-treated globin. Adding 2 or 4 μ*M* PhotoCORM produced ∼4 or 7.2 μ*M* CO-bound Mb; thus, per mole of PhotoCORM, approximately two of the three carbonyl ligands bind Mb (44). Addition of 10 μ*M* PhotoCORM produced CO saturation of Mb, yielding 12 μ*M* CO-Mb, similar to the effect observed by bubbling Mb with CO gas (Fig. 2A). Excess PhotoCORM (90 μ*M* PhotoCORM, 7 μ*M* reduced Mb) did not elicit spectral changes until the sample was exposed to UV (not shown). Thus, sodium dithionite, which triggers CO release from CORM-3 (40), does not cause CO release from [Mn(CO)_3_(tpa-κ^3^*N*)]^+^ and the Mb assay is a suitable method for CO quantification.

To fully release CO from the PhotoCORM and use the resulting molecule as a control (*i.e.*, an inactivated PhotoCORM or, more correctly, PhotoCORM exposed to UV light for 30 min with stirring to deplete CO [CO-depleted PhotoCORM]), a PhotoCORM stock solution (3 m*M*) was exposed to UV for up to 40 min with constant stirring to promote gas liberation to the atmosphere. Addition of PhotoCORM, previously subjected to illumination, to reduced Mb (4 μ*M* PhotoCORM and 12 μ*M* Mb) produced only 0.16 μ*M* CO-Mb, 1.3% of the total Mb (Fig. 2B). Illumination for 30–40 min did not reveal a typical heme-CO complex (Fig. 2B) (note difference in abscissa scales in Fig. 2A, B). Exposure of PhotoCORM to UV light for 30–40 min also promoted formation of a brownish insoluble precipitate (not shown) on the container wall, which may contribute to the spectral changes observed with PhotoCORM and Mb. PhotoCORM pre-exposed to UV (pre-illuminated PhotoCORM) and the supernatant of the CO-depleted PhotoCORM (30 min illumination) were used as control compounds.

### Activated PhotoCORM inhibits respiration of EC958 membranes

**FIG. 3.**
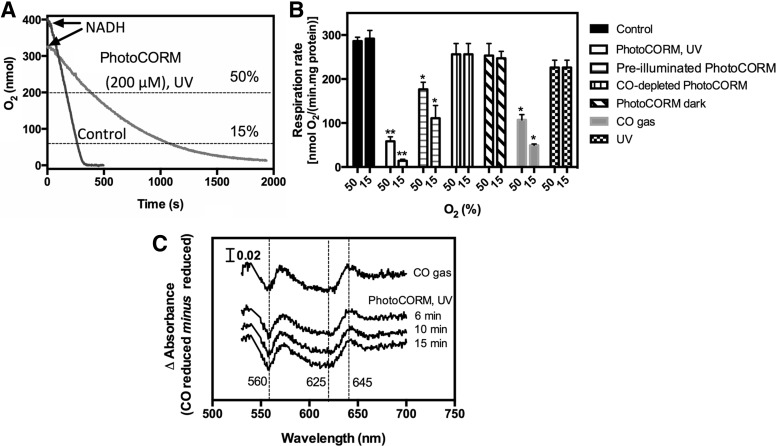
**Activated PhotoCORM inhibits respiration of EC958-purified membranes and releases CO to oxidases.** Isolated membranes from *Escherchia coli* EC958 were resuspended in Tris-HCl buffer (50 m*M*, pH 7.4). In **(A)**, are shown representative O_2_ electrode traces of O_2_ consumption in a closed chamber after adding NADH (*arrows*) to untreated membranes (control) or membranes exposed to UV light for 6 min in the presence of 200 μ*M* PhotoCORM (PhotoCORM, UV). The Figure also shows 50% and 15% air saturation (*dashed lines*) at which respiration rates were calculated. In **(B)**, are shown respiration rates at 50% and 15% air saturation from control and PhotoCORM, UV samples [as in **(A)**], and samples treated with PhotoCORM pre-exposed to UV light for 6 min (pre-illuminated PhotoCORM) or 30 min (to UV light for 30 min with constant stirring) or PhotoCORM kept in the dark (200 μ*M* final concentrations), followed by transfer to the closed chamber. A solution of CO (200 μ*M*) or an equivalent volume of water, followed by exposure to UV light for 6 min, was used as control. Bars represent standard deviation of at least three technical repeats of one representative biological repeat (**p < 0.0001; *p < 0.0005 with respect to the untreated control. In **(C)**, are shown difference spectra (globin plus PhotoCORM or CO *minus* globin, all reduced) of intact cells of strain EC958 (suspension OD ∼55) treated after reduction with sodium dithionite with either CO gas or 100 μ*M* PhotoCORM. Illumination was for 6–15 min as indicated. OD, optical density.

CO binding to respiratory oxidases and other heme proteins is generally assumed to be the principal mode of toxicity (28). However, CORM metal centers have also been implicated in the antibacterial activity of CORMs [reviewed in (64)]. To investigate inhibition of oxidase activity, bacterial membranes were treated with PhotoCORMs (200 μ*M*), exposed to UV, and then immediately transferred to a closed oxygen electrode chamber. Because NADH-supported respiration rates were not linear, they were calculated at both 50% and 15% of air saturation (Fig. 3A). Illuminated PhotoCORMs inhibited respiration compared with the untreated samples at 50% O_2_, an effect even more pronounced at low O_2_ tension (15%) (Fig. 3B). Membranes exposed to UV in the presence of PhotoCORMs were inhibited by 80% and 95% at 50% and 15% oxygen tensions, respectively, when compared with the untreated control, while membranes treated with pre-illuminated PhotoCORM were inhibited by 40% and 60% (Fig. 3B), probably due to CO loss to the atmosphere during the transfer of the pre-exposed PhotoCORM. Since PhotoCORM or CO-depleted PhotoCORM failed to inhibit membrane respiration in the dark, but CO gas (200 μ*M*) did (Fig. 3B), we deduce that inhibition of respiration by illuminated PhotoCORM (200 μ*M*) was directly related to CO release.

This was confirmed by direct spectroscopic examination of the oxidases in intact cells after treatment with PhotoCORM (Fig. 3C). Over 6–15 min after illumination, the spectral signatures were indistinguishable from the effects of bubbling the cells with CO gas. Most evident are features from the quinol oxidase, cytochrome *bd*; the peak near 645 nm is due to the CO-ligated ferrous cytochrome *d* and the trough centered at about 625 nm is due to bleaching of the cytochrome *d* absorbance. Features at 550–570 nm are due to *b*-type hemes.

The respiratory inhibition of membranes by pre-illuminated PhotoCORM (Fig. 3B) was surprising given that little CO was detected in the Mb assay (see above and Fig. 2B). This may be due to initial loss of two CO equivalents upon photoactivation, while the third requires a slow dark reaction (2). Further polarographic measurements were therefore carried out in an open chamber system to follow changes in respiration for longer times. Purified membranes, supplemented with NADH, reached a steady state at ∼10% O_2_ (Supplementary Fig. S2A). Adding pre-illuminated PhotoCORM (200 μ*M*) immediately inhibited respiration, reflected in a new higher steady state after 5 min. Similar effects were observed with subsequent aliquots of pre-illuminated PhotoCORM (Supplementary Fig. S2A, B). Since no effect was observed on adding nonexposed PhotoCORM (Supplementary Fig. S2A inset, B), and three subsequent aliquots of 200 μ*M* CO gas or pre-illuminated PhotoCORM caused comparable results (Supplementary Fig. S2C), we conclude that CO remaining in solution after illumination inhibits respiration.

We further investigated the effects of PhotoCORM on EC958 respiratory systems by measuring expression of genes encoding the two main terminal oxidases of *E. coli*, cytochromes *bo′* (*cyoA*) and *bd* (*cydA*). Both were only slightly downregulated by exposure to activated PhotoCORM and a slight downregulation was also seen following exposure to CO-depleted PhotoCORM (Supplementary Table S1).

### Activation of PhotoCORM in cultures reduces viability and inhibits growth of strain EC958

**FIG. 4.**
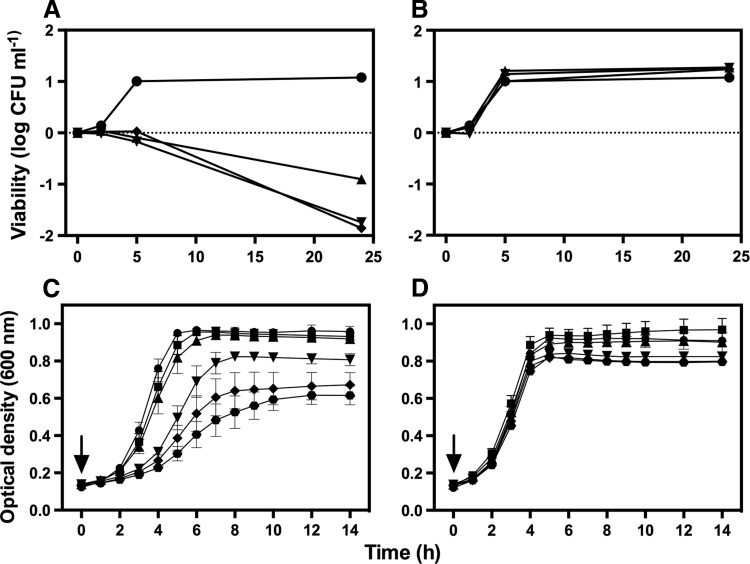
**PhotoCORM reduces viability and inhibits aerobic growth of** ***E. coli*** **EC958.** Cultures were grown in glucose minimal medium at 37°C, 200 rpm. (**A**) Shows quantification of CFU from cultures treated with 0 (●), 200 (▲), 350 (▼), and 500 μ*M* PhotoCORMs (**■**) pre-exposed to UV light for 6 min. In **(B)**, cultures were treated with PhotoCORMs as in **(A)**, but kept in the dark. Growth is represented as CFU/ml of treated cultures divided by the number of CFU/ml at time zero and expressed as log. **(C)** PhotoCORMs (at 0 [●], 50 [**■**], 100 [▲], 200 [▼], 300 (**♦**), and 500 [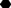] μ*M*) were added to cultures, followed by 6 min of exposure to UV light (365 nm). In **(D)**, cultures were treated with PhotoCORMs as in **(C)**, but kept in the dark. **(A)** and **(B)** are representative of three independent experiments. Compounds were added at time zero (*arrows*). Bars represent the standard error of three independent experiments. CFU, colony-forming unit.

Growth of *E. coli* K-12 MG1655 (a non-pathogenic strain) is inhibited only slightly by 500 μ*M* activated PhotoCORM in glucose minimal medium (44). However, for pathogenic strain EC958, also growing on glucose, all concentrations tested (200–500 μ*M*) reduced viability after illumination (Fig. 4A), but not in the dark (Fig. 4B). PhotoCORMs also inhibited growth significantly at concentrations of 200 μ*M* and above (Fig. 4C), but not in the dark (Fig. 4D). Although UV-pretreated PhotoCORM significantly inhibited respiration (Fig. 3 and Supplementary Fig. S2), adding 500 μ*M* of this compound did not inhibit growth (not shown), presumably because glucose supports nonrespiratory fermentative metabolism.

It is often assumed that the microbicidal toxicity of CORMs is due to released CO (46) that inhibits aerobic respiration by competing with O_2._ We therefore investigated PhotoCORM toxicity in anoxic cultures. Lower optical density (OD_600nm_) values were reached anoxically, so lower PhotoCORM concentrations were tested (Supplementary Fig. S3). A significant inhibitory effect was observed with 150 μ*M* light-activated PhotoCORM and the inhibition was slightly increased by addition of 200 or 250 μ*M* activated PhotoCORM (Supplementary Fig. S3A). As observed aerobically, cultures exposed to UV without PhotoCORM, treated with 250 μ*M* pre-illuminated PhotoCORM, or PhotoCORM kept in the dark were unaffected (Supplementary Fig. S3B). In conclusion, (a) the inhibitory effects of PhotoCORMs depend on light activation of the compound, but (b) the antimicrobial effect is independent of O_2_ and thus distinct from classical CO respiratory inhibition. Importantly, the effects of a CORM cannot always be attributed to heme binding.

### Activated PhotoCORM, but not CO gas, inhibits the aerobic growth of EC958

Since CO gas inhibited membrane respiration, we hypothesized that growth of EC958 cultures would be affected similarly. However, CO dissolved in culture medium even at 600 μ*M* (final concentration) was not toxic, yet adding 200 μ*M* PhotoCORM, followed by light activation, was clearly inhibitory (Supplementary Fig. S4). Rationalization of such results is difficult, but direct delivery of CO into bacteria by CORM internalization and the delivery of high CO concentrations have been suggested (11, 73).

### CO is released from PhotoCORMs in dense cell suspensions upon UV illumination

**FIG. 5.**
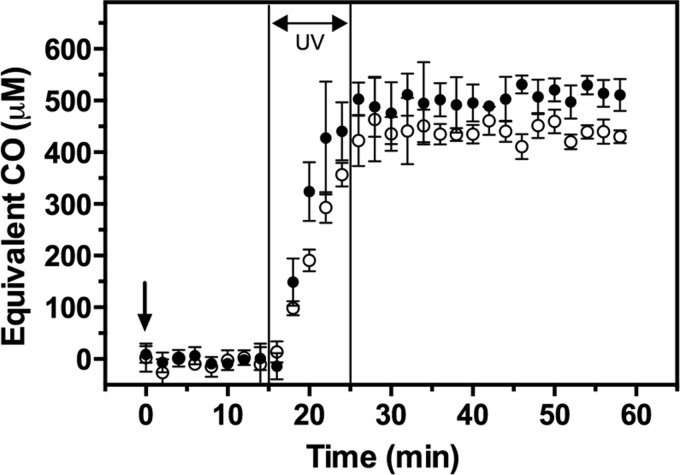
**PhotoCORM releases CO in thick cell suspensions upon exposure to UV.** Cell suspensions of *E. coli* EC958 (OD_600nm_ = 50) in glucose minimal medium were treated with PhotoCORMs (200 μ*M*) (●) and exposed to UV light for 10 min. Spectra of the headspace were measured every 2 min from 15 min before illumination, during the illumination period, and for 35 min afterward by Fourier transform infrared spectroscopy. For comparison, headspace measurements of PhotoCORM (200 μ*M*) illuminated for 10 min in minimal medium without bacteria were also measured (○). Error bars represent standard deviation of three independent experiments.

One potential drawback of using PhotoCORMs as an antibacterial agent might be releasing CO in turbid suspensions or tissues where UV may not penetrate. Gas-phase Fourier transform infrared (FT-IR) spectroscopy, measuring the intrinsic absorption of CO gas in the mid-infrared (400–4000 cm^−1^ [2500–25,000 nm]) (29, 50), was exploited to follow CO release from PhotoCORM (200 μ*M*) in a constantly stirred suspension of EC958 (OD_600nm_≈50) exposed to UV for 10 min. The equivalent CO detected in the headspace of the flask containing the cell suspension was ∼430 μ*M* (Fig. 5) [∼2 mol CO per mol PhotoCORM, in agreement with the Mb assay (Fig. 2A)]. In culture medium lacking cells, a slightly higher CO concentration was detected in the headspace (∼500 μ*M* equivalent CO) (Fig. 5). This is unlikely to be because CO is trapped by cells and unable to reach the gas phase since total heme content determined in the cell suspension was only 2.21 μ*M* (not shown). Thus, only very high cell concentrations or tissue density might limit UV penetration.

### CO from activated PhotoCORMs reaches the bacterial cytoplasm, but Mn is not accumulated in EC958 cells

Figure 3C showed that CO from activated PhotoCORMs reaches membrane oxidases, but to investigate cytoplasmic access, we used a non-pathogenic laboratory strain, MG1655, overexpressing a heterologous globin—the truncated hemoglobin (Ctb) from *Campylobacter jejuni* (1). The cytoplasmic globin sink traps CO released from the PhotoCORM, and the formation of CO-bound Ctb can be visualized in intact cells by dual-wavelength spectroscopy (68). Although Ctb has been extensively studied (68), no absorbance coefficient for the Soret region of the CO difference spectrum (CO reduced *minus* reduced) is available for *in vivo* quantitation of the CO-Ctb adduct. Therefore, known concentrations of Ctb, quantified from A_280_ measurements, were used to prepare CO difference spectra and ΔA (422–447 nm) plotted against concentration, giving an absorbance coefficient of 44 × 10^3^ *M*^−1^ s^−1^. Second, hemochrome (alkaline pyridine, reduced *minus* oxidized) assays on the protein were performed (51); assuming a 1:1 ratio of heme B:protein, we derived an absorbance coefficient of 43 × 10^3^ *M*^−1^ s^−1^.

**FIG. 6.**
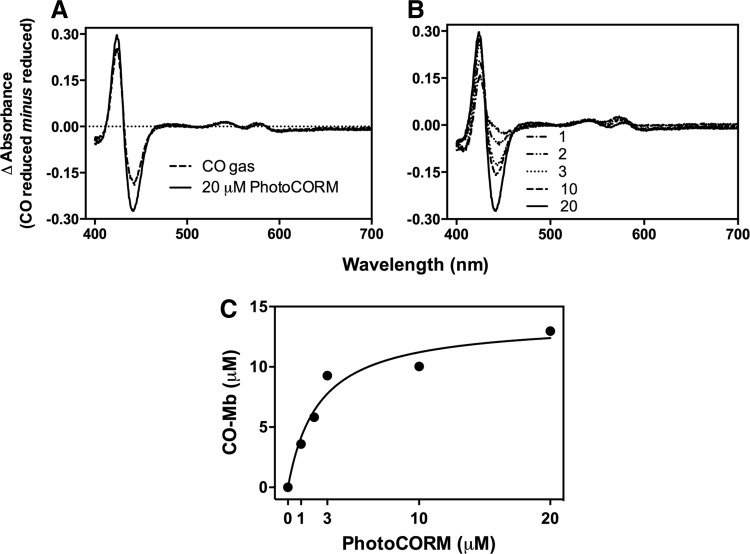
**Intracellular formation of CO-bound bacterial globin from activated PhotoCORM.** In **(A)**, *E. coli* MG1655 cell suspensions overexpressing globin (Ctb) were reduced by addition of glucose (15 m*M*) and then bubbled with CO gas to saturation or treated with 20 μ*M* PhotoCORMs and exposed to UV light (365 nm) for 6 min. In **(B)**, cell suspensions were treated with increasing concentrations of PhotoCORMs and exposed to UV for 6 min. Difference in absorbance (CO reduced *minus* reduced) was plotted (numbers indicate PhotoCORM [μ*M*]). In **(C)**, the amount of CO-Ctb formed was calculated from **(B)** and plotted against PhotoCORM concentration.

Ctb-expressing cells in buffer were supplemented with glucose to promote respiration, thereby removing O_2_ and providing reducing equivalents for globin reduction essential for CO binding. A cell suspension containing ∼13 μ*M* ferrous Ctb was either bubbled with CO or treated with PhotoCORM (20 μ*M*), then exposed to UV. Difference spectra (CO reduced *minus* reduced) revealed that both CO gas and activated PhotoCORM generated 10–13 μ*M* CO-bound Ctb (Fig. 6A). When Ctb-expressing cells were treated with increasing concentrations of PhotoCORMs and exposed to UV (Fig. 6B), the concentration of PhotoCORM correlated with the amount of intracellular CO-bound Ctb (Fig. 6C).

To investigate whether CO might be delivered to hemes directly from accumulated PhotoCORMs, intracellular Mn was assayed by inductively coupled plasma mass spectometry (ICP-MS) in cells grown with 50 μ*M* PhotoCORM, either in the dark or activated *in situ*. The intracellular Mn concentration detected, even after 80 min, was not significantly higher than in samples without the PhotoCORM, regardless of incubation time or whether the cultures were exposed to UV (Supplementary Fig. S5A). When culture supernatants and cell washes (to capture loosely bound Mn) were analyzed, ∼50 μ*M* Mn remained (Supplementary Fig. S5B, C) showing that Mn from the PhotoCORM is not significantly accumulated. This conclusion appears at variance from a study with strain MG1655 (44) where, on prolonged dark incubation with the PhotoCORM, a late abrupt uptake of the compound was observed. However, (i) that strain was grown in Evans medium, not defined minimal medium, (ii) dimethylsulfoxide was present as solvent, and (iii) strain MG1655 consistently accumulates more metal than does EC958 (results not shown). Nevertheless, as positive controls, we tested EC958 with CORM-3 and CORM-401. CORM-3 is accumulated to high levels (11), and CORM-401 reaches millimolar levels in strain MG1655 (L.K. Wareham and R.K. Poole, in preparation). EC958 also accumulated both CORM-3 and CORM-401 to high levels (2370 μ*M* Ru and 1430 μ*M* Mn, respectively; not shown). We conclude that PhotoCORM is not significantly accumulated by strain EC958, although other CORMs are.

It is possible that cells internalize the PhotoCORM that is then rapidly exported (after CO release following activation). However, the partition coefficients (log *P*) determined for activated PhotoCORM, the PhotoCORM kept in the dark, and the CO-depleted PhotoCORM show that the compounds are not hydrophobic and probably unable to passively cross biological membranes (Table 1). It should be noted that the genome of EC958 encodes drug export systems that could transport PhotoCORMs or its products (66).

**Table 1.** Lipophilicity and Partition Coefficient (Log *P*) For PhotoCORM and the CO-Depleted Form

*Compound*	*Aqueous phase (a)*^a^	*Organic phase (o)*^a^	*log* P *(o/a)*
PhotoCORM, UV	99.0	1.00	−2.00
PhotoCORM dark	99.5	0.50	−2.30
CO-depleted PhotoCORM	96.8	3.20	−1.50

^a^ Mn (determined by ICP-MS) recovered from aqueous and organic phases, expressed as a percentage of the total found after partition.

CO-depleted PhotoCORM, PhotoCORM exposed to UV light for 30 min with stirring to deplete CO; PhotoCORM, photoactivable carbon monoxide-releasing molecule.

### Effects of PhotoCORMs on transport gene expression

To investigate possible transport mechanisms for the PhotoCORM, we performed real-time polymerase chain reaction (RT-PCR) on PhotoCORM-treated cells and examined transport systems for Mn. These, however, probably act on naked Mn ions in the +II oxidation state, possibly [Mn(H_2_O)_6_]^2+^ in aqueous solution, whereas the PhotoCORM is in the +I state and embedded by the tpa ligand and the three CO ligands. Indeed, *mntH*, encoding an Mn importer (34), was downregulated twofold on treatment with PhotoCORMs and illumination (Supplementary Table S1) and to a lesser extent by CO-depleted PhotoCORMs, consistent with the lack of significant Mn accumulation. In *E. coli* strain, MG1655, aerobic CO exposure elicits extensive downregulation of the enterochelin genes required for high-affinity iron uptake (L.K. Wareham and R.K. Poole, submitted). Therefore, transcriptional changes in *entE* were investigated (Supplementary Table S1). Although *entE* was upregulated about fourfold in response to UV-activated PhotoCORM (Supplementary Table S1), it was also 5.5-fold elevated in response to CO-depleted PhotoCORM. In pathogenic *E. coli*, uptake of heme as an iron source is facilitated by the ChuA receptor (65); in this study, *chuA (*operonic with *chuS*, encoding HO) was upregulated by PhotoCORM (>2-fold) and CO-depleted PhotoCORM (3.5-fold). The modest upregulation of iron acquisition systems is currently unexplained.

### The combination of activated PhotoCORM and H_2_O_2_ is highly toxic to EC958 cultures

**FIG. 7.**
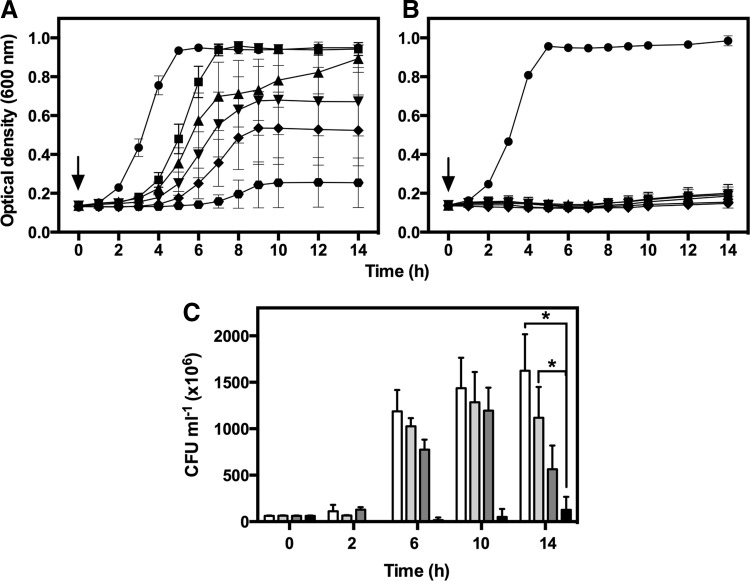
**Combination of activated PhotoCORM with H_2_O_2_ impairs growth of EC958.** Cultures were grown in glucose minimal medium at 37°C, 200 rpm. In **(A)**, cultures were added with 0 (●), 6 (**■**), 7 (▲), 8 (▼), 9 (♦), and 10 (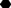) mM H_2_O_2_. In (**B**), control (no additions) (●) and cultures treated with PhotoCORM (100 μM) plus 4 (**■**), 5 (▲), 6 (▼), 7 (**♦**), and 8 (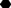) m*M* H_2_O_2_ were exposed to UV for 6 min. Compounds were added at time zero (*arrows*). In **(C)**, over the same time scale as in **(A)** and **(B)**, cell viability is shown in cultures exposed to UV for 6 min in the absence (*white bars*) or presence of PhotoCORM (100 μ*M*) (*light gray bars*), H_2_O_2_ (4 m*M*) (*dark gray bars*), or a combination of both compounds (*black bars*). Samples taken immediately before treatment were recorded as time zero. Bars represent the standard error of at least three independent experiments. Student's test was used to compare the viability of cultures treated with H_2_O_2_ and PhotoCORM at 14 h to each of the other conditions, *p < 0.05. H_2_O_2_, hydrogen peroxide.

The failure of CO gas to mimic the toxicity of activated PhotoCORM in EC958 cultures led us to hypothesize that the Mn center, which undergoes oxidation state changes upon UV-activated loss of CO ligands (2), might also be involved in the toxicity together with, or perhaps instead of, the released CO. As Mn was not accumulated intracellularly (Supplementary Fig. S5), interaction of the activated PhotoCORM with reactive extracellular molecules was considered. H_2_O_2_ endogenously generated in respiration can diffuse from cells (33), so we tested whether the presence of external H_2_O_2_ increased toxicity of the activated PhotoCORM. EC958 cultures were highly resistant to H_2_O_2_ (Fig. 7A), 10 m*M* H_2_O_2_ being required for almost total inhibition. However, when cultures containing a subinhibitory concentration of PhotoCORM (100 μ*M*) were treated with H_2_O_2_ and exposed to UV, only 4 m*M* H_2_O_2_ completely impaired growth (Fig. 7B) and decreased viability to zero after 1 h (Fig. 7C).

**FIG. 8.**
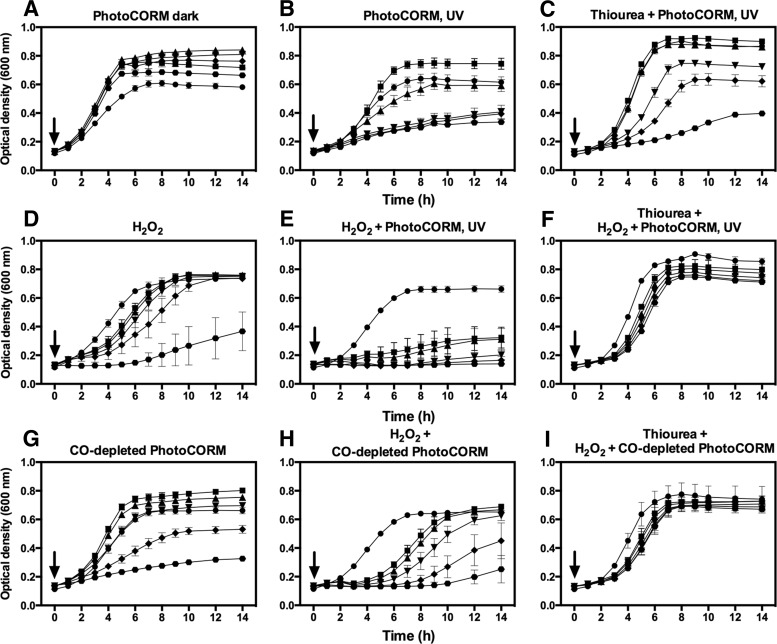
**Toxicity from the combination of activated PhotoCORM and H_2_O_2_ is alleviated by thiourea in cultures of EC958.** Cultures were grown in Fe-depleted glucose minimal medium at 37°C, 200 rpm. **(A)**: 0 (●), 50 (**■**), 100 (▲), 200 (▼), 300 (♦), and 500 (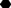) μ*M* PhotoCORMs. In **(B)**, PhotoCORM was added as in **(A)**, followed by being exposed to UV light (365 nm) for 6 min. **(C)** is as **(B)**, but thiourea (80 m*M*) was added to cultures before the treatment with PhotoCORM. In **(D)**, 0 (●), 6 (**■**), 7 (▲), 8 (▼), 9 (♦), and 10 (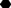) m*M* H_2_O_2_ was added. In **(E)**, cultures treated with PhotoCORM (100 μ*M*) 4 (**□**), 5 (▲), 6 (▼), 7 (♦), and 8 (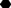) m*M* H_2_O_2_ were exposed to UV for 6 min and compared with an untreated control (●). **(F)** is as **(E)**, but thiourea (80 m*M*) was added to all cultures before the addition of PhotoCORM and H_2_O_2_. **(G)** is as **(A)**, but CO-depleted PhotoCORM was added instead. In **(H)**, cultures treated with CO-depleted PhotoCORM (100 μ*M*) were supplemented with 4 (**■**), 5 (▲), 6 (▼), 7 (♦), and 8 (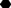) m*M* H_2_O_2_ and compared with an untreated control (●). **(I)** is as **(H)**, but thiourea (80 m*M*) was added to all cultures before the addition of CO-depleted PhotoCORM and H_2_O_2_. Compounds were added at time zero (*arrows*). Bars represent the standard error of at least three independent experiments.

Since H_2_O_2_ reacts avidly with iron, generating hydroxyl radicals, and glucose minimal medium contains a high concentration of FeCl_3_ (∼20 μ*M*) (16), we tested PhotoCORMs and H_2_O_2_ in Fe-depleted medium. Inhibition of the growth caused by activated PhotoCORMs (PhotoCORM, UV) in Fe-depleted (Fig. 8B) and Fe-replete media was similar (compare Figs. 4C and 8B). However, cultures containing PhotoCORMs not exposed to UV grew faster and reached higher ODs than the untreated control (Fig. 8A), a phenomenon that we did not observe in iron-replete conditions (compare Figs. 4D and 8A).

Cultures grown in Fe-depleted medium were substantially more resistant to H_2_O_2_ (6–10 m*M*) (Fig. 8D) than those grown in Fe-replete medium (compare Figs. 8D with 7A), attributable to the production of hydroxyl radicals in the Fenton reaction. Strikingly, combining activated PhotoCORM (100 μ*M*) with H_2_O_2_ (4–8 m*M*) produced remarkable inhibition of growth in Fe-depleted medium (Fig. 8E), although it was slightly less pronounced compared with iron-replete medium (compare Figs. 8E and 7B). Cultures treated with nonactivated PhotoCORMs or pre-illuminated PhotoCORMs (100 μ*M* each) in combination with H_2_O_2_ (6 m*M*) were not significantly inhibited (Supplementary Fig. S6).

Since the toxicity of PhotoCORMs combined with H_2_O_2_ depended on the activation of the compound *in situ* (Fig. 8E), and growth was not inhibited by addition of CO gas combined with H_2_O_2_ (not shown), it seemed plausible that H_2_O_2_ directly interacts with the Mn-containing compound that results from the UV-promoted release of CO. To explore this, we tested the effect of CO-depleted PhotoCORM alone and in combination with H_2_O_2_. The toxicity of the inactivated compound was significant at the highest concentrations tested (300 and 500 μ*M*), while lower concentrations failed to cause inhibition (Fig. 8G). On the other hand, addition of CO-depleted PhotoCORM (100 μ*M*) plus H_2_O_2_ produced detrimental effects at all concentrations (4–8 m*M* H_2_O_2_) (Fig. 8H). However, the toxicity was marginally lower than that observed with PhotoCORM, UV (compare Fig. 8H and E). Thus, light activation of PhotoCORMs *in situ* promotes, but is not essential for, the synergy with H_2_O_2_. To further investigate the interaction between PhotoCORM and H_2_O_2_, we tested whether manganese sulfate as a source of Mn^II^, alone or with H_2_O_2_ and/or CO gas, inhibited growth following UV illumination, but no growth inhibition was observed (not shown).

We tested the hypothesis that PhotoCORM toxicity is related to endogenous generation of oxidative stress, as proposed for some CORMs (61). Very little change of expression was seen in genes responsible for oxidative stress defense (*katG* and *sodA)* in response to PhotoCORM or CO-depleted PhotoCORM alone. As expected, *katG* was substantially upregulated by H_2_O_2_ (55-fold, expressed as log_2_ in Supplementary Table S1), but was upregulated less by H_2_O_2_ in combination with PhotoCORM (14-fold), perhaps due to depletion of H_2_O_2_ in hydroxyl formation (18) (see below).

Finally, we examined whether the PhotoCORM induces membrane damage, as inferred from massive upregulation of the *spy* gene by CORMs such as CORM-3 (11, 74). EC958 cells treated with 150 μ*M* PhotoCORM, then illuminated, did not show *spy* upregulation (Supplementary Table S1). Interestingly, however, combining H_2_O_2_ with PhotoCORM and illumination elicited extensive *spy* upregulation (∼14-fold) compared with H_2_O_2_ (∼6-fold) or PhotoCORM alone, consistent with generation of reactive oxygen species on reaction of the Mn in CO-depleted CORM with H_2_O_2_.

### Toxicity of activated PhotoCORM against EC958 is partially alleviated by the hydroxyl scavenger, thiourea

We hypothesized that Mn from PhotoCORM reacts with H_2_O_2_ to produce, as in the case of Fe, hydroxyl radicals. Indeed, thiourea (80 m*M*) added before activated PhotoCORM or CO-depleted PhotoCORM plus H_2_O_2_ (Fig. 8F, I) protected cultures from inhibition. This is persuasive evidence for the formation of hydroxyl radicals by reaction of PhotoCORM with H_2_O_2_. Cultures containing activated PhotoCORM (without H_2_O_2_) were only marginally protected by thiourea (Fig. 8C). Since the activated PhotoCORM inhibits growth anaerobically (Supplementary Fig. S3A), reaction with reactive oxygen species cannot be the sole explanation.

### Activated PhotoCORM and Mn react with H_2_O_2_ producing hydroxyl radicals

**FIG. 9.**
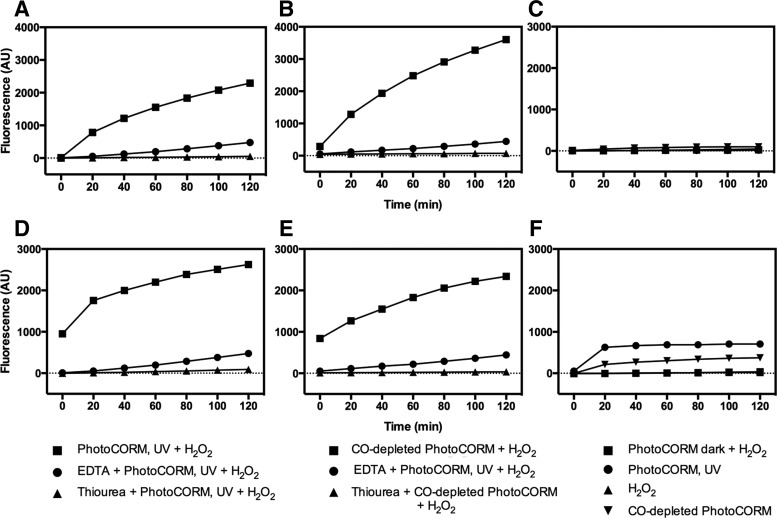
**The combination of PhotoCORM and H_2_O_2_ produces hydroxyl radicals.** Fluorescence was measured in glucose minimal medium **(A**–**C)** or Fe-depleted glucose minimal medium **(D**–**F)**. Samples containing PhotoCORMs were exposed to UV light for 6 min (PhotoCORM, UV) or kept in the dark (PhotoCORM dark). PhotoCORM and CO-depleted PhotoCORM final concentrations were 10 μ*M*. HPF (5 μ*M*) was added after the activation of the PhotoCORM, the addition of PhotoCORM dark or CO-depleted PhotoCORM, and before the addition of H_2_O_2_ (300 μ*M*). EDTA or thiourea (5 and 3 m*M* final concentration, respectively) was added to samples before the addition of the PhotoCORM or CO-depleted PhotoCORM. EDTA, ethylenediaminetetraacetic acid; HPF, 3′-(*p*-hydroxyphenyl) fluorescein.

To test whether activated PhotoCORM reacts with H_2_O_2_, generating hydroxyl radicals, the dye 3′-(*p*-hydroxyphenyl) fluorescein (HPF), which specifically detects hydroxyl radicals, but does not react with H_2_O_2_, was used (58). A rapid sustained increase in fluorescence revealed the production of hydroxyl radicals in samples containing PhotoCORM (10 μ*M*) and H_2_O_2_ (300 μ*M*), and then light activated (Fig. 9 and Supplementary Fig. S7). Addition of ethylenediaminetetraacetic acid (EDTA, 5 m*M*) drastically decreased the fluorescence, presumably by chelating Mn (4). Combination of nonactivated PhotoCORM (PhotoCORM dark) with H_2_O_2_ failed to produce hydroxyl radicals (Fig. 9C, F). MnSO_4_ alone did not generate hydroxyl radicals, but on illumination in the presence of H_2_O_2_, significant hydroxyl generation occurred (Supplementary Fig. S7), approaching the levels seen with illuminated PhotoCORM at 120 min. Thus, the toxicity observed in cultures arises directly from the interaction of the Mn center with peroxide once the PhotoCORM loses CO. Interestingly, some fluorescence was detected in Fe-depleted minimal medium from activated PhotoCORM and CO-depleted PhotoCORM in the absence of H_2_O_2_ (Fig. 9F). However, as thiourea failed to prevent fluorescence (not shown), the basis of this phenomenon remains unknown.

### Potentiation by PhotoCORM of the antimicrobial effect of an antibiotic

Strain EC958 is characterized by multiple antibiotic resistance (48). When bacteriological testing indicates appropriate susceptibility to the drug, doxycycline, a member of the tetracycline family, may be used to treat infections, including those caused by gram negatives such as *E. coli*. However, strain EC958 is relatively resistant to this antibiotic; growth studies in liquid medium showed a minimal inhibitory concentration of around 96 μg/ml, which was reduced to 24 μg/ml after treatment with 200 μ*M* PhotoCORM and illumination (Supplementary Fig. S8).

## Discussion

Most CORMs studied biologically release CO *via* ligand exchange reactions (25), but trigger mechanisms may be employed, including enzymatic cleavage (54), magnetic heating of CORM-loaded magnetite nanoparticles (30), and light-induced CO release (49, 53, 56, 57). In this study, we report the activities of an Mn PhotoCORM in far more detail than has been previously been achieved (44, 70).

Activation of CO release by UV illumination produces a compound that is more effective at reducing bacterial growth and viability than is CO gas at higher concentrations. Such findings have sometimes been attributed to high levels of intracellular CORM accumulation (11) and consequent localized CO delivery to target sites. However, the PhotoCORM is not measurably accumulated in this pathogenic strain, so CO is presumably liberated outside bacteria and the toxic effects are, in part, due to facile diffusion of CO to intracellular targets.

Nevertheless, the view that CO alone explains the toxicity of CORMs is oversimplistic. Bacteria demonstrate multiple transcriptomic changes to CORM-3 that cannot be understood in terms of known CO biochemistry [*e.g.*, (11, 73)], even bacteria lacking hemes are inhibited by CORM-3 and make transcriptomic responses (74). It is striking that ruthenium CORMs display much higher toxicity (11, 24, 73) than CO gas (72) or the Mn CORM, CORM-401 (10) (L.K. Wareham and R.K. Poole, in preparation), and the present PhotoCORM. Indeed, other ruthenium compounds are accumulated with toxic consequences even though they are not CORMs [*e.g.*, (31, 32)] and, in lysozyme, Ru(II)(CO)_2_-protein adducts formed at a histidine residue release CO (7). The relative lack of toxicity of Mn CORMs may prove valuable in clinical settings.

Even CORMs that do not release CO (inactivated or CO-depleted CORMs) can exert toxicity and alter gene regulation (39). In this study, we show that CO-depleted PhotoCORM retains biological activity, including the ability, like the native PhotoCORM, to react with H_2_O_2_, generating hydroxyl radicals. For example, *spy* regulation (and by inference membrane damage) results from the reaction of CO-depleted CORM with H_2_O_2_. The H_2_O_2_ concentration required to produce a lethal combination with PhotoCORM is well below the level of H_2_O_2_ that is itself growth inhibitory. It is unlikely that endogenously generated H_2_O_2_ could diffuse from cells in concentrations sufficient to mimic the combined effect of exogenous H_2_O_2_ and PhotoCORM (21). High H_2_O_2_ concentrations inactivate iron enzymes and iron–sulfur dehydratases (59, 60), but a 15-fold increase in H_2_O_2_ production, representing an unrealistic 45% of cellular oxygen consumption (22) would be needed to generate even 8 μ*M* intracellular H_2_O_2_, which is not bactericidal (23). Thus, while endogenously generated H_2_O_2_ is insufficient to augment the bactericidal activity of PhotoCORM, the required concentrations could easily be administered in certain settings where surface sterilization is required as in topical and odontogenic infections.

**FIG. 10.**
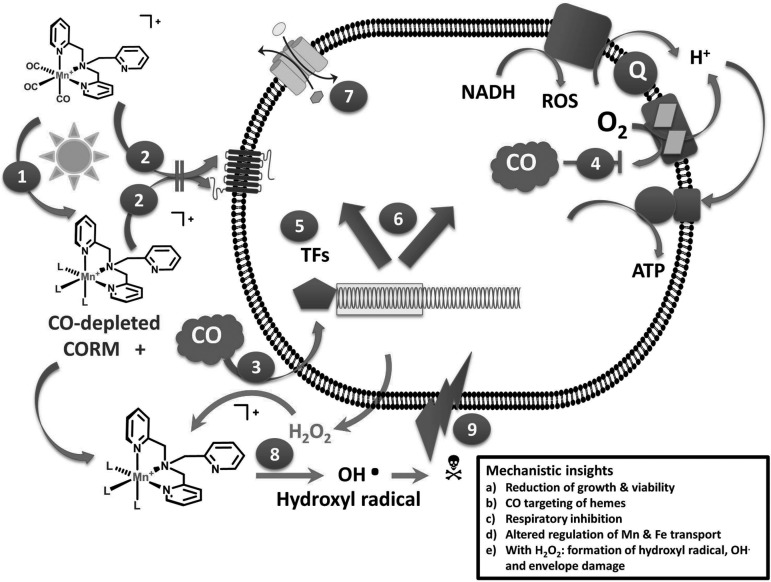
**Schematic visualization of the activities of PhotoCORM against** ***E. coli*** **strain EC958.** Light activation of the PhotoCORM at 365 nm 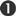 leads to release of the CO ligands from the manganese coordination sphere. The resulting Mn complex is not transported inward 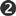, while CO enters the cell *via* passive diffusion 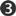, and inhibits NADH-supported respiration 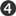 by competing with oxygen, thereby restricting ATP generation. ROS may be formed. CO binds to cytoplasmic heme proteins (not shown) and is sensed by TFs 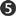, resulting in transcriptional changes 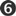 in, for example, genes involved in metal acquisition 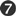. Following the loss of CO, the compound reacts with hydrogen peroxide, exogenous, or metabolism derived 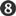, forming cytotoxic products such as hydroxyl radicals 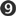 that perturb membrane integrity. The symbol L indicates the diverse solvent- or biomolecule-derived ligands that take the position of the released carbon monoxide. ROS, reactive oxygen species; TF, transcription factor.

CORMs were developed for safe and controlled CO delivery (17, 19, 36) and were only later investigated for antimicrobial activity. It is important to recognize that for no CORM—even those that have been extensively studied for many years—do we have a complete picture of the mechanisms of toxicity. This is due to the complicated speciation of the resulting metal–coligand fragment (CO-depleted or iCORM = inactivated CORM) that can bind constituents of the medium and/or the cell in place of the CO released and, in the case of transition metal-based CORMs, undergo oxidation state changes based on the redox state of the system. Thus, only by understanding the toxicity of a well-characterized compound can CORMs more suitable for clinical use be designed. In this study, we extensively studied a PhotoCORM and draw the following conclusions: (i) UV illumination, even in thick suspensions (and, by extension, tissues), releases two CO ligands that access intracellular heme targets, thereby inhibiting aerobic respiration, even though the CORM manganese cannot be detected intracellularly. (ii) The inhibition of anaerobic growth by activated PhotoCORM suggests mechanisms of toxicity unrelated to classical aerobic respiration. (iii) Illuminated PhotoCORM is a more effective antimicrobial agent than CO or the nonactivated species. (iv) PhotoCORM and subtoxic concentrations of H_2_O_2_ are synergistic in their antimicrobial effects and generate hydroxyl radicals. (v) CO-depleted PhotoCORM also generates, with H_2_O_2_, toxic species that perturb membrane integrity. (vi) The doxycycline resistance of this pathogenic strain is, in part, overcome by coapplication of the activated PhotoCORM. (vii) Finally, our data do not support models of CORM toxicity that invoke generation of other reactive oxygen species or intracellular metal accumulation as key players. These findings are summarized in Figure 10.

Only by understanding the multifaceted aspects of CORM reactivity with biological systems can the potential for controlled spatial and temporal CO release be realized. PhotoCORMs, in particular, may have translational applications in topical treatments or where a photolyzing source can access the desired sites of application, as in the oral cavity or urinary tract [for examples of PACT, see refs. (27, 41)]. Indeed, a catheter light guide for prostate or bladder surgery incorporates a waveguide for light transmission (Patent application US20100016844 A1). Thus, PhotoCORMs warrant much more detailed investigation and understanding, potentially in combinatorial therapies with antibiotics (Supplementary Fig. S8).

## Materials and Methods

### Reagents

Synthesis of PhotoCORM [Mn(CO)_3_(tpa-κ^3^*N*)]Br is described in Nagel *et al.* (44). Aqueous stock solutions (10 m*M*) were kept in the dark for up to 24 h at 4°C. Pre-illuminated PhotoCORM was obtained by illuminating the PhotoCORM stock for 6 min with a UV lamp (UVITEC Cambridge, 365 nm) placed 3 cm above the sample. CO-depleted PhotoCORM was prepared by exposing 1 ml PhotoCORM (3 m*M*) to UV for 30 min with stirring. CO-saturated solutions were prepared by bubbling CO gas into water or glucose minimal medium for 30 min and used immediately. Other chemicals were from Sigma.

### Growth conditions

Bacteria were stored on Luria Broth (LB) (Miller; Formedium) plates at 4°C. For *E. coli* EC958, plates were supplemented with ampicillin (100 μg/ml). EC958 was used throughout unless otherwise stated. Starter cultures were grown in LB broth overnight at 37°C, 200 rpm. After centrifugation, cells were resuspended in minimal medium (16) or Fe-depleted minimal medium (lacking the FeCl_3_ present in minimal medium) with glucose (20 m*M*) as sole carbon source and used to inoculate (at 3% [v/v]) fresh medium (∼0.12 OD_600nm_). For aerobic growth, 2.5 ml cultures were prepared in 5-ml plastic containers and, when indicated, treated with PhotoCORM, CO-depleted PhotoCORM, H_2_O_2_, and/or thiourea. To activate PhotoCORM, open containers were exposed to UV from above (3 cm) (PhotoCORM, UV samples). An aliquot (200 μl) was transferred to 96-well plates and incubated for 14 h at 37°C, 200 rpm, in a Sunrise™ microplate reader (TECAN). For anaerobic growth, 7-ml vials were filled to the brim with medium and statically incubated at 37°C. For FT-IR measurements, LB cultures were grown to an OD_600nm_ of 1.0. Cells were harvested by centrifugation and resuspended in glucose minimal medium (∼10 ml [50 OD_600_]).

### Viability studies

Samples (20 μl) were serially diluted in phosphate-buffered saline. Eight aliquots (10 μl each) from each dilution were inoculated onto LB plates and incubated overnight at 37°C, and colony-forming units were determined and averaged.

### FT-IR measurements

Infrared spectra of the headspace were recorded using a Matteson Research Series FT-IR spectrometer equipped with a DTGS detector at a resolution of 0.4 cm^−1^. The cell suspension was transferred to a custom flask equipped with two gas-tight taps and a third port equipped with a rubber seal for purging and reagent addition. This was then attached to a custom IR gas cuvette (CaF_2_ windows, 14.5 cm path length) housed within the IR spectrometer. An airtight peristaltic pump (7 l/h flow rate) circulated the culture headspace into the gas cuvette and back into the flask, bringing vapor phase to equilibrium within 2 min. Each IR spectrum was accumulated for eight scans (1 min each). The resulting transmission spectra were converted to absorbance using an independent background measurement of laboratory air recorded before each series of measurements. Spectra were baseline corrected. Before measurements, the system was purged with a nitrogen flow for 30 min with continuous stirring. PhotoCORM was added (200 μ*M*) and the system was purged for a further 10 min in darkness. IR spectra of the headspace were recorded every 2 min to test for nonphotolytic CO release. After removal of the nitrogen purge line, the sample was illuminated at 365 nm for 10 min through the vessel wall with continuous stirring and cycling of the headspace. Spectra were recorded every 2 min during illumination and for 35 min afterward to follow CO release to the headspace. CO in the gas phase was quantified by comparing the integrated absorbance of the R-branch of the CO fundamental (2142–2235 cm^−1^) with the absorption cross sections from the HITRAN2012 database (55).

### Expression of Ctb in *E. coli*

For overexpression of *C. jejuni* hemoglobin (Ctb) in *E. coli*, strain MG1655 lacking the flavohemoglobin (*hmp* mutant) and transformed with plasmid pLW1 [*ctb* under control of an arabinose-inducible promoter (67)] [strain RKP3920, (1)] or an empty vector [RKP3919 (1)] was grown aerobically in LB supplemented with ampicillin (100 μg/ml), 5-aminolevulinic acid (500 μ*M*), and FeCl_3_ (12 μ*M*). When 0.3 OD_600nm_ was reached, 0.02% arabinose (v/v) was added and cultures were incubated for 4 h and stored overnight at 4°C.

### Optical spectrometry

CO release from the PhotoCORM was assessed *in vitro* by the Mb assay (9). A solution of Mb (12 μ*M*) in 0.1 *M* phosphate buffer (pH 7.4) was reduced with a few grains of sodium dithionite. In a 3-ml cuvette, 2 ml of the reduced Mb solution was bubbled with CO gas for 3 min or treated with PhotoCORM and exposed to UV or treated with pre-illuminated PhotoCORM. For spectroscopy of oxidases *in vivo*, difference spectra (CO reduced, or reduced and treated with PhotoCORM, *minus* reduced) were recorded with a Johnson Foundation SDB3 dual-wavelength scanning instrument (26). For studying intracellular formation of CO-Ctb, difference spectra (CO reduced *minus* reduced) were recorded using an Olis RSM1000 spectrophotometer. Cultures overexpressing Ctb or carrying an empty vector were harvested and resuspended in 10 ml Tris-HCl 50 m*M* (pH 7.4) and the OD_600_ was standardized. Reduction was achieved by adding glucose (15 m*M*) (63), and O_2_ consumption was followed polarographically in a closed chamber. Upon depletion of O_2_, the lid was removed to allow air diffusion into the sample and the O_2_ levels recorded for a further 1 h with stirring. Reduced samples of the Ctb-expressing strain (RKP 3920) were treated with increasing concentrations of PhotoCORMs and exposed to UV light, and changes in the spectra were recorded immediately. As a control, a reduced sample of the same strain was bubbled with CO gas for 3 min. A strain carrying the empty vector (RKP 3919) was used to obtain the absolute spectrum of intracellular Ctb and assure reduction before PhotoCORM addition. Difference spectra (CO reduced *minus* reduced) were plotted. Intracellular concentration of Ctb was determined by reducing the Ctb-expressing samples with sodium dithionite, followed by bubbling with CO gas. The extinction coefficient of the Ctb difference spectrum (CO reduced *minus* reduced) is 43.5 m*M*/cm. Heme was determined as in Poole *et al.* (51).

For whole cell spectroscopy, cells were grown to mid-exponential phase and suspended after washing to an approximate OD_600_ of 55. Difference spectra were taken of cells reduced by dithionite and incubated with [Mn(CO)3(tpa-κ3N)]Br (100 μ*M*) at room temperature *minus* reduced cells alone using the SDB3 spectrophotometer. During incubation (6, 10, and 15 min), the cell suspensions were illuminated at 365 nm using a UV hand lamp (UVIlite LF-206LS, 6 W; UVItec Ltd.).

### Isolation of bacterial membranes

This was based on Poole and Haddock (52). Cultures were grown until they reached ∼1.5 OD_600_, then centrifuged, and the pellet resuspended in membrane isolation buffer (52). Protein concentration was determined by the Markwell assay (38).

### Respiration measurements

For assays in a closed system (73), purified membranes resuspended in Tris-HCl buffer (50 m*M*, pH 7.4) (2 ml in a 7-ml vial) were illuminated from above with a UV lamp at 365 nm (distance ∼3 cm) in the presence or absence of PhotoCORM (200 μ*M*) and the sample immediately transferred to the O_2_ electrode chamber to measure respiration. Controls were performed by addition of PhotoCORM in the dark, pre-illuminated PhotoCORM, CO-depleted PhotoCORM, or CO gas from CO-saturated water, all at final concentrations of 200 μ*M*. Assays in an open electrode system were performed in the same chamber, but lacking the sealing cap (12). A steady state was achieved on adding membranes to buffer (2 ml), followed by NADH (2.5 m*M*) to promote respiration. At steady state, three subsequent additions of PhotoCORM pre-exposed to UV or PhotoCORM kept in the dark were performed (200 μ*M* final concentration each). Respiration rates were calculated from the measured inward oxygen diffusion rates (12) and normalized by protein content.

### Mn uptake by growing cells

Aerobic cultures of EC958 at mid-exponential phase (∼0.4 OD_600_) were treated with PhotoCORMs (50 μ*M*) and either kept in the dark or exposed to UV. Samples were analyzed as in Davidge *et al.* (11) using literature values for single cell dry mass and volume (45).

### Determination of the lipophilicity of PhotoCORM and its derivatives

A modification of the shake-flask method was utilized (69). Briefly, glucose minimal medium was used as the aqueous phase and *n*-hexane (presaturated with medium) as the organic phase. The hexane layer was then isolated. PhotoCORM was dissolved in medium at 10 m*M* and exposed to UV or kept in the dark. CO-depleted PhotoCORM was prepared by dissolving PhotoCORM in medium at 3 m*M*, followed by exposure to UV for 30 min with constant stirring. An equal volume of n-hexane and medium containing activated PhotoCORM, PhotoCORM in the dark, or CO-depleted PhotoCORM were mixed and left to shake overnight at 37°C. After separation, each layer was sampled. The *n*-hexane was evaporated at room temperature and the volume replaced with aqueous solution. The amount of Mn in both layers was determined by ICP-MS.

### Hydroxyl radical production

The assay was performed in glucose minimal medium or Fe-depleted glucose minimal medium. Samples (3 ml medium) containing PhotoCORM were exposed to UV or kept in the dark. PhotoCORM and CO-depleted PhotoCORM final concentrations were 10 μ*M*. The fluorescent reporter dye HPF (Invitrogen; 5 μ*M*) was used for detecting hydroxyl radicals. The probe was added after activation of the PhotoCORM, the addition of PhotoCORM dark, CO-depleted PhotoCORM or MnSO_4_, and before the addition of H_2_O_2_. EDTA or thiourea (5 and 3 m*M* final, respectively) was added to samples before the PhotoCORM or CO-depleted PhotoCORM. Fluorescence intensity was measured using an F-2500 fluorescence spectrophotometer (Hitachi) (490 nm excitation, 515 nm emission).

### Real-time polymerase chain reaction

Exponential phase cultures were treated with PhotoCORM (150 μ*M*), CO-depleted PhotoCORM (150 μ*M*), or H_2_O_2_ (2 m*M*), alone or in combination. Following treatment, cultures were incubated at 37°C for 10 min with shaking at 200 rpm and exposed where indicated to UV. Aliquots of culture were removed to RNAprotect (Qiagen) and total RNA was prepared using an RNeasy RNA purification kit (Qiagen) and quantified using a NanoDrop 1000 spectrophotometer (Thermo Scientific). RT-PCR was done in an Mx3005P Thermocycler (Agilent Technologies) using the Brilliant III Ultra-Fast SYBR Green qRT-PCRMaster Mix kit (Agilent Technologies). A genomic DNA dilution series was used to correct for differences in primer amplification efficiencies, and the housekeeping gene *gyrA* was used for normalization. The mean log_2_ ratios of individual gene expression relative to that in unstressed cells were calculated (*n* = 3 ± SD). The primer sets used were *spy*, 5′ CTGCACTGTTTGTTGCCTCTAC 3′ and 5′ AACTTGCCTTTGTGGTGCAT3′; *katG*, 5′ CCATAACACCACAGCCACTG 3′ and 5′ AGTTGATTTGGCCACCAGTC 3′; *sodA*, 5′ TGAGCTATACCCTGCCATCC 3′ and 5′ TCTGATGGTGTTTGGTGTGG 3′; *cyoA*, 5′ TTGCAGGCACTGTATTGCTC 3′ and 5′ CCAAATGCCGTCAGTATCAG 3′; *cydA*, 5′ TAGTCGAACTGTCGCGCTTA 3′ and 5′ GAGGACGTAGACCGTTTCCA 3′; *chuA*, 5′ CAATTTACTTCGTTGCGTTTGA 3′ and 5′ CGTAACGGTCATGGTTTCAGTA 3′; *entE*, 5′ AAGAGTTTGCCCGTCGCTAT 3′ and 5′ AGTCAGAATGTCGGTCAGTGG 3′; *mntH*, 5′ AACTATCGCGTTGAGAGTAGCA 3′ and 5′ CAATCCCTAGTTTGGCAGAGAG 3′; and *gyrA*, 5′ GGTACACCGTCGCGTACTTT 3′ and 5′ TACCGATTACGTCACCAACG 3′.

## Supplementary Material

Supplemental data

Supplemental data

Supplemental data

Supplemental data

Supplemental data

Supplemental data

Supplemental data

Supplemental data

Supplemental data

## Abbreviations Used

CFU: colony-forming unit
CO-depleted PhotoCORM: PhotoCORM exposed to UV light for 30 min with stirring to deplete CO
CO-Mb: CO-bound myoglobin
CORM: carbon monoxide-releasing molecule
EDTA: ethylenediaminetetraacetic acid
FT-IR: Fourier transform infrared
HO: heme oxygenases
HPF: 3′-(*p*-hydroxyphenyl) fluorescein
ICP-MS: inductively coupled plasma mass spectometry
Mb: myoglobin
OD: optical density
PACT: photoactivated chemotherapy
PhotoCORM: photoactivable carbon monoxide-releasing molecule
ROS: reactive oxygen species
RT-PCR: real-time polymerase chain reaction
TF: transcription factor
UPEC: uropathogenic *E. coli*
UTI: urinary tract infection
