# Supplementary material for: Antimicrobial Activity of the Manganese Photoactivated Carbon Monoxide-Releasing Molecule [Mn(CO)3(tpa-κ3N)]+ Against a Pathogenic Escherichia coli that Causes Urinary Infections
Source: Antioxid Redox Signal. 2016 May 10;24(14):765–80. doi: 10.1089/ars.2015.6484 (PMC4876522; doi:10.1089/ars.2015.6484)
Supplement: Supplemental data [file Supp_Figure1.pdf]

Supplementary Data

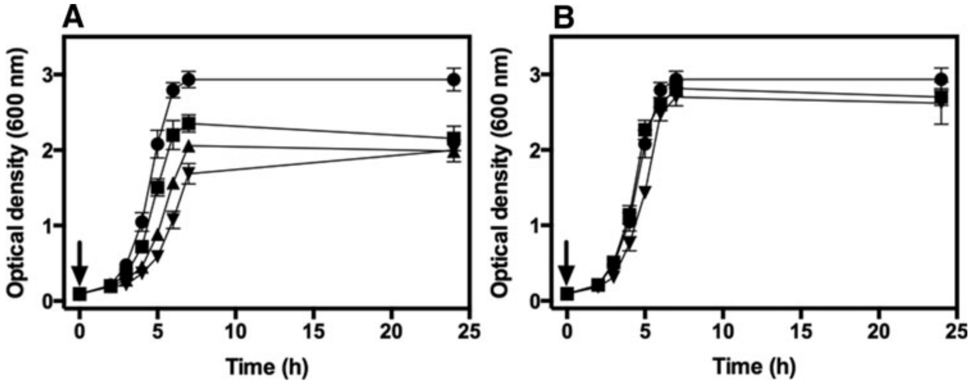

SUPPLEMENTARY FIG. S1. Inhibition of the growth of strain EC958 by PhotoCORM is dependent on activation by exposure to UV. Cultures were grown in glucose minimal medium at 37°C, 200 rpm. In **(A)**, cultures were exposed to UV (365 nm) for 3 (■), 6 (▲), or 10 min (▼) after adding 200 *μM* PhotoCORM and compared with an untreated culture (●). In **(B)**, cultures illuminated at 365 nm for 10 min in the absence of PhotoCORM (■) or treated with 200 *μM* PhotoCORM and kept in the dark (▼) were compared with the control (●). *Arrows* show addition of PhotoCORM. Bars represent the standard error of three independent experiments. PhotoCORM, photoactivable carbon monoxide-releasing molecule.
