# Supplementary material for: Antimicrobial Activity of the Manganese Photoactivated Carbon Monoxide-Releasing Molecule [Mn(CO)3(tpa-κ3N)]+ Against a Pathogenic Escherichia coli that Causes Urinary Infections
Source: Antioxid Redox Signal. 2016 May 10;24(14):765–80. doi: 10.1089/ars.2015.6484 (PMC4876522; doi:10.1089/ars.2015.6484)
Supplement: Supplemental data [file Supp_Figure2.pdf]

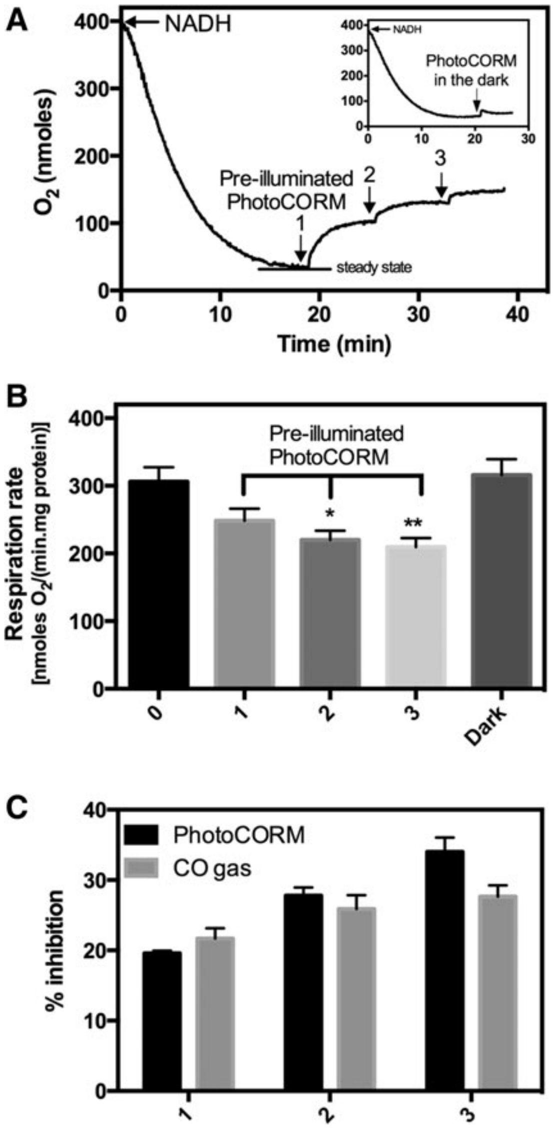

SUPPLEMENTARY FIG. S2. PhotoCORM pre-exposed to UV light inhibits membrane respiration. Purified membranes from *Escherichia coli* EC958 were resuspended in Tris-HCl buffer (50 m*M*, pH 7.4), and O_2 consumption, upon addition of NADH, was polarographically recorded in an open chamber. In **(A)**, when the respiration rate reached steady state, three consecutive aliquots of pre-UV-exposed PhotoCORM for 6 min (200 μM final concentration each) were added (1, 2, 3). An aliquot of PhotoCORM (200 μM) kept in the dark was added as a control (*inset*). In **(B)**, respiration rates were calculated from the traces in **(A)** when respiration reached steady state and normalized by the protein content. In **(C)**, inhibition of membrane respiration upon addition of three subsequent aliquots of PhotoCORM or CO gas (200 μM final concentration) was compared and respiration rates calculated and expressed as percentage inhibition. 1, 2, and 3 represent three subsequent additions of pre-illuminated PhotoCORM or CO gas. Figures are representative of two biological experiments performed independently. Bars represent standard deviation of at least three technical repeats of one representative biological repeat (**$p < 0.032$, *$p < 0.04$, with respect to the untreated control).
