# Supplementary material for: Antimicrobial Activity of the Manganese Photoactivated Carbon Monoxide-Releasing Molecule [Mn(CO)3(tpa-κ3N)]+ Against a Pathogenic Escherichia coli that Causes Urinary Infections
Source: Antioxid Redox Signal. 2016 May 10;24(14):765–80. doi: 10.1089/ars.2015.6484 (PMC4876522; doi:10.1089/ars.2015.6484)
Supplement: Supplemental data [file Supp_Table1.pdf]

Supplementary Table S1. Levels of Gene Expression Assessed by Real-Time Polymerase Chain Reaction in Response to PhotoCORM and Other Conditions

	Genes involved in							
	Encoding respiratory oxidases		*Iron and manganese acquisition*			*Responses to reactive oxygen species*		*Response to membrane damage*
Sample condition	cyo	Cyd	mntH	chuA	entE	katG	sodA	Spy
Photocorm, UV	−0.63 ± 0.13	−0.41 ± 0.25[a]	−0.94 ± 0.17	+1.4 ± 0.25	+2.2 ± 0.38	−0.60 ± 0.34[a]	+0.75 ± 0.76[a]	−0.19 ± 0.24[a]
CO-depleted PhotoCORM	−0.31 ± 0.058	−0.42 ± 0.10	−0.67 ± 0.089	+2.2 ± 0.37	+2.9 ± 0.28	n.d.	n.d.	n.d.
PhotoCORM dark	−0.15 ± 0.11	−0.24 ± 0.070	+0.20 ± 0.045[a]	+0.97 ± 0.068[a]	+1.2 ± 0.068[a]	n.d.	n.d.	n.d.
H_2O_2	n.d.	n.d.	n.d.	n.d.	n.d.	+5.7 ± 0.99	+2.1 ± 0.20[a]	+2.7 ± 0.21[a]
H_2O_2 + photoCORM, UV	n.d.	n.d.	n.d.	n.d.	n.d.	+3.7 ± 1.0	+1.9 ± 0.28[a]	+3.7 ± 0.71[a]

Cultures of EC958 were treated with PhotoCORM or CO-depleted PhotoCORM (150 *μM*), alone or in combination with H_2O_2 (2 m*M*). A culture was also treated with 2 m*M* H_2O_2 alone as a control. The mean $\log_2$ ratios of individual gene expression relative to the housekeeping gene *gyrA* were then compared with unstressed cells ($n = 3 \pm$ SD, except "[a]" $n = 2$). + and − indicate upregulation and downregulation, respectively. For example, a two-fold increase is given as +1, and a two-fold decrease is given as −1.

CO-depleted PhotoCORM, photocorm exposed to UV light for 30 min with stirring to deplete CO; H_2O_2, hydrogen peroxide; n.d., not determined; PhotoCORM, photoactivable carbon monoxide-releasing molecule.
