# Supplementary material for: Antimicrobial Activity of the Manganese Photoactivated Carbon Monoxide-Releasing Molecule [Mn(CO)3(tpa-κ3N)]+ Against a Pathogenic Escherichia coli that Causes Urinary Infections
Source: Antioxid Redox Signal. 2016 May 10;24(14):765–80. doi: 10.1089/ars.2015.6484 (PMC4876522; doi:10.1089/ars.2015.6484)
Supplement: Supplemental data [file Supp_Figure3.pdf]

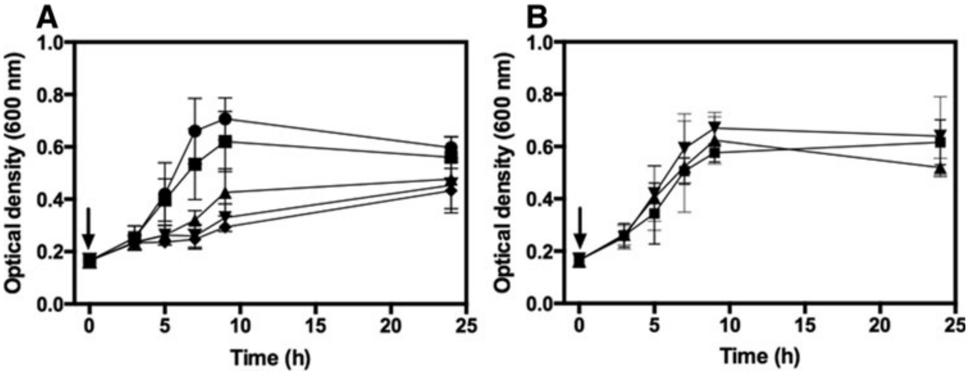

SUPPLEMENTARY FIG. S3. PhotoCORM inhibits anaerobic growth of *E. coli* EC958. Cultures were grown in glucose minimal medium at 37°C statically in the absence of oxygen. In **(A)**, 0 (●), 50 (■), 150 (▲), 200 (▼), and 250 *μM* PhotoCORMs (■) were added to cultures, then exposed to UV light for 6 min. In **(B)**, cultures exposed to UV light in the absence of PhotoCORM (▼), treated with 250 *μM* PhotoCORM and kept in the dark (■), or treated with 250 *μM* PhotoCORM pre-exposed to UV light for 6 min (▲) are shown. Compounds were added at time zero (*arrows*). Bars represent the standard error of three independent experiments.
