# Supplementary material for: Antimicrobial Activity of the Manganese Photoactivated Carbon Monoxide-Releasing Molecule [Mn(CO)3(tpa-κ3N)]+ Against a Pathogenic Escherichia coli that Causes Urinary Infections
Source: Antioxid Redox Signal. 2016 May 10;24(14):765–80. doi: 10.1089/ars.2015.6484 (PMC4876522; doi:10.1089/ars.2015.6484)
Supplement: Supplemental data [file Supp_Figure4.pdf]

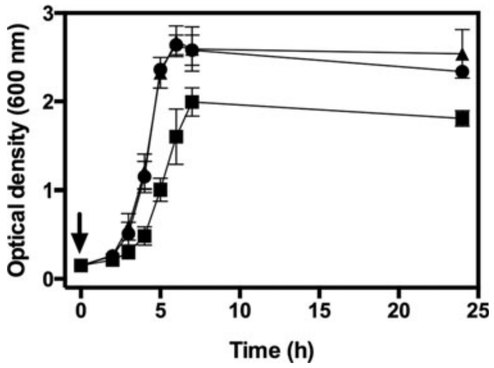

SUPPLEMENTARY FIG. S4. PhotoCORM, but not CO gas, inhibits the aerobic growth of EC958. Cultures were incubated at 37°C, 200 rpm. PhotoCORM 200 μM (■) or 600 μM CO gas (from CO saturated medium) (▲) was added to cultures at time zero (*arrow*) and exposed to UV light for 6 min (365 nm). (●) Represents the nontreated control. Bars are the standard deviation of three independent experiments.
