# Supplementary material for: Antimicrobial Activity of the Manganese Photoactivated Carbon Monoxide-Releasing Molecule [Mn(CO)3(tpa-κ3N)]+ Against a Pathogenic Escherichia coli that Causes Urinary Infections
Source: Antioxid Redox Signal. 2016 May 10;24(14):765–80. doi: 10.1089/ars.2015.6484 (PMC4876522; doi:10.1089/ars.2015.6484)
Supplement: Supplemental data [file Supp_Figure5.pdf]

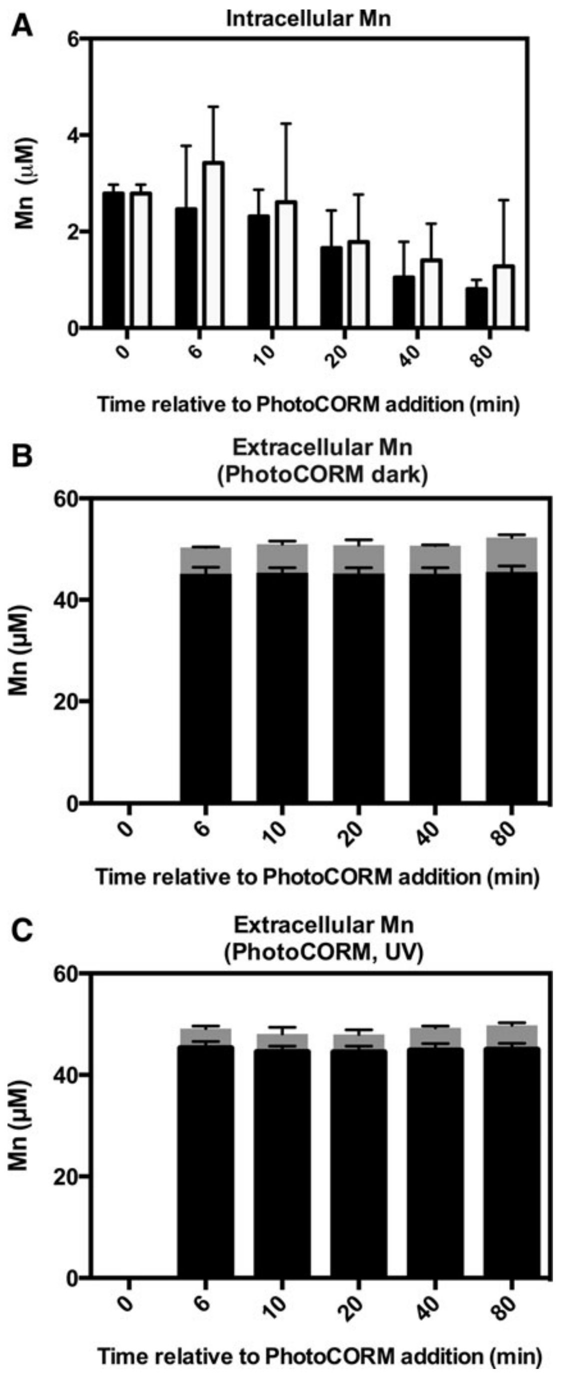

SUPPLEMENTARY FIG. S5. Mn is not accumulated intracellularly in EC958 cultures exposed to PhotoCORM. Cultures were grown in the presence of PhotoCORM (50 *μM*). In **(A)**, intracellular Mn was quantified from cultures containing PhotoCORM kept in the dark (*black bars*) or exposed to UV light (365 nm) for 6 min (*white bars*). Time zero shows the intracellular Mn concentration before addition of PhotoCORM, representing normal intracellular pools. In **(B)**, extracellular Mn was quantified in the supernatants of cultures kept in the dark after addition of the PhotoCORM (*black bars*) and washes of cell pellets to remove loosely bound Mn (*gray bars*). **(C)** is as **(B)**, but from cultures exposed to UV light. Samples were taken at different time points and Mn determined by inductively coupled plasma mass spectrometry (ICP-MS). Bars are the standard deviation of three independent experiments.
