# Supplementary material for: Antimicrobial Activity of the Manganese Photoactivated Carbon Monoxide-Releasing Molecule [Mn(CO)3(tpa-κ3N)]+ Against a Pathogenic Escherichia coli that Causes Urinary Infections
Source: Antioxid Redox Signal. 2016 May 10;24(14):765–80. doi: 10.1089/ars.2015.6484 (PMC4876522; doi:10.1089/ars.2015.6484)
Supplement: Supplemental data [file Supp_Figure6.pdf]

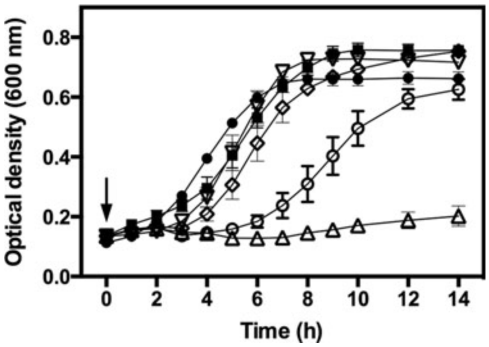

SUPPLEMENTARY FIG. S6. The toxicity of PhotoCORM combined with H_2O_2 depends on the activation of PhotoCORM in the presence of peroxide. Cultures were grown in Fe-free glucose minimal medium at 37°C, 200 rpm. Shown are untreated control (●); H_2O_2 (6 m*M*) (■); PhotoCORM (100 *μM*) plus H_2O_2 (6 m*M*), followed by UV (△); PhotoCORM (100 *μM*) and H_2O_2 (6 m*M*) (no exposure to UV) (▽); PhotoCORM (100 *μM*) pre-exposed to UV plus H_2O_2 (6 m*M*) (◇); and co-depleted PhotoCORM (100 *μM*) plus H_2O_2 (6 m*M*) (○). Compounds were added at time zero (*arrows*). Bars represent the standard error of three independent experiments. H_2O_2, hydrogen peroxide.
