# Supplementary material for: Antimicrobial Activity of the Manganese Photoactivated Carbon Monoxide-Releasing Molecule [Mn(CO)3(tpa-κ3N)]+ Against a Pathogenic Escherichia coli that Causes Urinary Infections
Source: Antioxid Redox Signal. 2016 May 10;24(14):765–80. doi: 10.1089/ars.2015.6484 (PMC4876522; doi:10.1089/ars.2015.6484)
Supplement: Supplemental data [file Supp_Figure7.pdf]

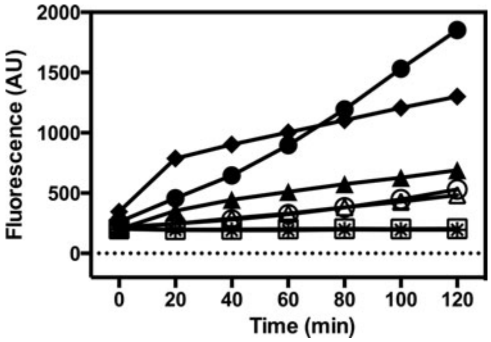

SUPPLEMENTARY FIG. S7. Hydroxyl radical production by metal ions either alone or in combination with H_2O_2. Fluorescence was measured in glucose minimal media. $MnSO_4$ concentration was always $10\,\mu M$, H_2O_2 was $5\,mM$, and HPF was $5\,\mu M$. Shown are samples containing $MnSO_4$, followed by UV and HPF, before addition of H_2O_2 (●); $MnSO_4$ with no exposure to UV, followed by HPF, before addition of H_2O_2 (○); H_2O_2, followed by UV and HPF (▲); H_2O_2 with no exposure to UV and HPF (△). As controls, fluorescence of HPF in media alone (*); and $MnSO_4$ plus HPF without H_2O_2 (□) were also measured. Ferrous perchlorate ($100\,\mu M$), followed by the addition of HPF and H_2O_2 (◆), was used as a positive control for hydroxyl radical production. HPF, 3′-(*p*-hydroxyphenyl) fluorescein.
