# Supplementary material for: Antimicrobial Activity of the Manganese Photoactivated Carbon Monoxide-Releasing Molecule [Mn(CO)3(tpa-κ3N)]+ Against a Pathogenic Escherichia coli that Causes Urinary Infections
Source: Antioxid Redox Signal. 2016 May 10;24(14):765–80. doi: 10.1089/ars.2015.6484 (PMC4876522; doi:10.1089/ars.2015.6484)
Supplement: Supplemental data [file Supp_Figure8.pdf]

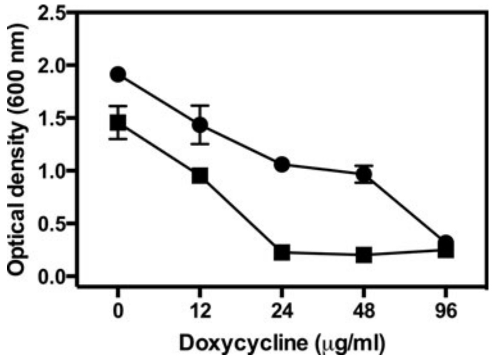

SUPPLEMENTARY FIG. S8. PhotoCORM potentiates the antimicrobial effect of the antibiotic doxycycline. Cultures were treated with doxycycline alone (●) or plus PhotoCORM (■) for 24 h and the final optical densities were measured.
